# Dramatic Changes in Thiopurine Metabolite Levels in a Patient With Inflammatory Bowel Disease Treated With Tirzepatide for Weight Loss

**DOI:** 10.14309/crj.0000000000001544

**Published:** 2024-11-06

**Authors:** Jeremy A. Klein, Joëlle St-Pierre, David Choi, Jacqueline Lopez, David T. Rubin

**Affiliations:** 1 Inflammatory Bowel Disease Center, University of Chicago Medicine, Chicago, IL

**Keywords:** thiopurines, GLP-1 agonist, J pouch, ulcerative colitis

## Abstract

Thiopurines can be used to maintain remission in patients with inflammatory bowel disease. Thiopurines require regular blood count monitoring and, in specific patients, thiopurine metabolites for assessment of optimization and safety. We present the case of a 42-year-old woman with ulcerative colitis postcolectomy and ileal pouch-anal anastomosis with subsequent antibiotic-resistant diffuse pouchitis and prepouch ileitis. She was in stable remission with thiopurine monotherapy. Following tirzepatide initiation, she experienced elevated liver enzymes associated with a significant increase in thiopurine metabolite levels. This case underlines the importance of monitoring metabolite levels in patients with inflammatory bowel disease initiated on glucagon-like peptide 1-targeted therapies.

## INTRODUCTION

Inflammatory bowel disease (IBD) is a chronic inflammatory condition that includes ulcerative colitis (UC) and Crohn's disease. There are many effective therapies for induction and maintenance of remission in IBD, including thiopurines.^1,2^ For medically refractory cases of UC or when neoplasia develops, total proctocolectomy with ileal pouch-anal anastomosis (IPAA) is often performed. However, IPAAs are associated with risks of pouchitis and Crohn's disease along with postcolectomy immune panenteropathy.^3^

6-mercaptopurine (6-MP) has variable bioavailability due to first-pass metabolism and genetic variations.^4^ As such, thiopurine methyltransferase activity is tested before initiation.^5^ During treatment with thiopurines, blood counts and liver enzymes are assessed. In some patients where concerns of therapeutic efficacy or toxicity arise, thiopurine metabolites (6-thioguanine [6-TG] and 6-methylmercaptopurine [6-MMP]) are assessed. This provides information regarding metabolism and opportunities for dose adjustment to maximize the efficacy and reduce myelotoxicity and hepatotoxicity.^6^

Tirzepatide (a dual glucose-dependent insulinotropic polypeptide (GIP) and glucagon-like peptide 1 receptor agonist [GLP-1 RA]) was approved by the US Food and Drug Administration for type 2 diabetes in May 2022, and for weight loss in November 2023.^7,8^ GLP-1 RAs cause delayed gastric emptying, inhibit small intestine motility, and increase gastric residue rates, which may affect pharmacokinetics of coingested medications.^9^ A recent systematic review did not find a clinically significant impact on the metabolism of common medications (statins, angiotensin-converting enzyme inhibitors, warfarin, among others) with concomitant GLP-1 RA, but the interaction of GLP-RA with thiopurines has not been reported.^10^

Here, we discuss a 42-year-old woman with a history of ulcerative pancolitis complicated by diffuse pouchitis with prepouch ileitis receiving 6-MP. She was prescribed tirzepatide for weight loss and subsequently presented with hepatotoxicity and significant increase in thiopurine metabolite levels.

## CASE REPORT

We present a 42-year-old woman with a 16-year history of IBD. She was diagnosed with UC at the age of 26 years and treated with mesalamine, followed by the combination of infliximab and thiopurine. At the age of 36 years, she developed flat low-grade dysplasia and underwent a laparoscopic total proctocolectomy followed by standard 3-stage IPAA.

**Figure 1.**
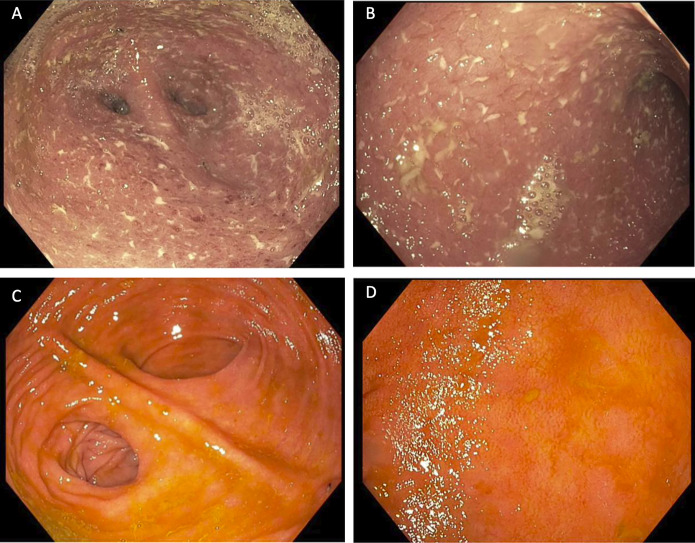
Pouchoscopy before and after 6-MP treatment. Endoscopic findings consistent with endoscopically severe pouchitis (A) and prepouch ileitis (B). Pouchoscopy after starting 6-MP demonstrating endoscopic remission in pouch (C) and prepouch ileum (D). 6-MP, six-mercaptopurine.

Three years later, she developed abdominal pain, 5–7 loose bowel movements per day with nocturnal symptoms, and 4.5 kg weight loss. Her symptoms did not respond to empiric antibiotics. Pouchoscopy and biopsy revealed diffuse pouchitis and significant prepouch ileitis (Figure 1). A diagnosis of postcolectomy panenteritis was considered, but upper endoscopy was not performed.^3^ Her symptoms rapidly improved on prednisone, and she started 75 mg of 6-MP daily, after thiopurine methyltransferase activity was confirmed normal. She was confirmed in symptomatic remission after 11 weeks, and subsequent pouchoscopy showed endoscopic healing (Figure 1). Thiopurine metabolites were measured at various times with 6-TG ranging from 209 to 281 pmol/8 × 10^8^ red blood cells (RBC) and 6-MMP from 3,711 to 3,932 pmol/8 × 10^8^ RBC. While in remission, she remained in good health but noted a 15 kg weight gain.

**Figure 2.**
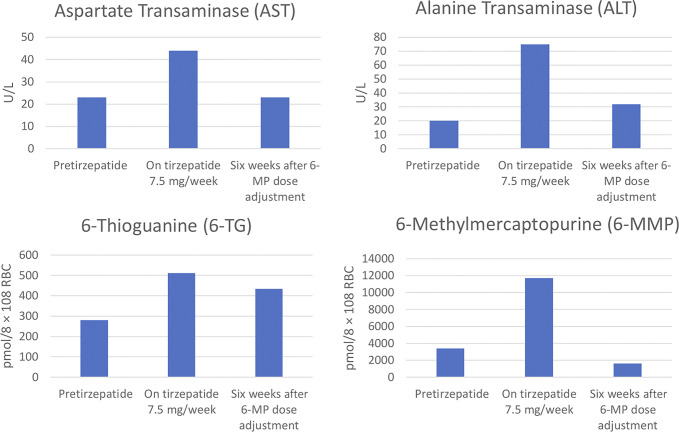
Aspartate aminotransferase, alanine aminotransferase, and thiopurine metabolite levels pretirzepatide, while on maintenance tirzepatide (7.5 mg/wk), and 6 weeks after 6-MP dose decrease from 75 to 50 mg. 6-MP, six-mercaptopurine.

She started tirzepatide 2.5 mg per week in October 2023 for indications of elevated body mass index (31 kg/m^2^) and hypercholesterolemia, and uptitrated to 7.5 mg in December 2023. She had a 14.1 kg weight loss (body mass index decreased to 25.8 kg/m^2^) on tirzepatide with no changes to her medications or medical history. However, routine laboratory tests in February 2024 identified transaminase elevations of aspartate aminotransferase (AST) 44 U/L, alanine aminotransferase (ALT) 75 U/L from normal prior (AST 21 U/L, ALT 19 U/L). At that time, 6-TG was elevated to 512 pmol/8 × 10^8^ red blood cells (RBC) and 6-MMP elevated to 11,704 pmol/8 × 10^8^ RBC (Figure 2). C-reactive protein and complete blood count remained normal.

She was advised to hold 6-MP for 1 week (she continued tirzepatide), followed by a decrease in dosing from 75 to 50 mg. Repeat blood work 6 weeks later showed resolution of transaminitis (AST 23 U/L, ALT 32 U/L) along with improvement of thiopurine metabolite levels (6-TG 434 pmol/8 × 10^8^ RBC and 6-MMP 1,608 pmol/8 × 10^8^ RBC) (Figure 2). She subsequently continued 50 mg daily of 6-MP. Her bowel remained in symptomatic remission.

## DISCUSSION

GLP-1 RA use is increasing given the growing population with diabetes and obesity. In addition to promoting weight loss, GLP-1 RAs are linked to early satiety and dose-dependent deceleration of gastric transit times.^9^ Tirzepatide is a dual GLP-1 and GIP RA that is more potent than monotargeted GLP-1 RA.^11^ It is administered through subcutaneous injection, has 80% bioavailability, and a half-life of 5 days.^12^ Moreover, it has a greater dose-dependent transient effect on gastric emptying that is seen after the first dose when compared with typical GLP-1 RAs.^13^ Of note, GIP does not affect gastric emptying due to the lack of receptors on gastric and small bowel tissue.^14^

There are no current studies showing the effect of tirzepatide on thiopurine metabolism. Oral absorption of 6-MP is incomplete and variable with average bioavailability of 16%.^15^ 6-MP has a high volume of distribution and, therefore, serum 6-MP concentration can increase with adipose tissue loss.^16^ Thiopurine metabolites follow the first-order elimination kinetics and are excreted primarily in the urine.^17^ Tirzepatide is unlikely to influence hepatic metabolism of thiopurines. We hypothesize that the slower gastric and duodenal transit time (mainly due to the GLP-1 RA effects) allowed for increased 6-MP absorption. We also propose that anti-inflammatory properties of GLP-1 RA may augment bowel healing and facilitate improved thiopurine absorption.^18^ In addition, the patient's weight loss (14.1 kg) on GLP-1 therapy may affect weight-based dosing. At tirzepatide initiation, dosing was 0.89 mg/kg compared with 1.07 mg/kg after weight loss. However, this remains within safe weight-based 6-MP dosing (1.0–1.5 mg/kg).^19^

Although this report is based on a single patient, extra caution should be taken for patients on GLP-1 RA. We propose adopting a more proactive approach by repeating metabolite levels 1 month after GLP-1 RA initiation. Larger studies are needed to confirm these observations and to determine whether they are broadly representative of diverse patient groups. This case report details how GLP-1 RA affects thiopurine metabolite levels. Owing to widespread use of GLP-1 RA for diabetes and weight loss, it is important that patients on concomitant thiopurine therapy have closer monitoring of metabolite levels to avoid adverse effects.

## DISCLOSURES

Author contributions: DT Rubin: manuscript conception; DT Rubin, J. St-Pierre, JA Klein, and J. Lopez: data analysis; DT Rubin, J. St-Pierre, JA Klein, D. Choi, and J. Lopez: manuscript draft; DT. Rubin and JA Klein: final edits. DT. Rubin is the article guarantor.

Financial disclosure: JA Klein, J. St-Pierre, J. Lopez: no conflicts or disclosures. D. Choi: has served on the speaker bureau for Janssen Pharmaceuticals, Eli Lilly. Has served as a consultant to Bristol Myers Squibb, and Boehringer Ingelheim, AbbVie, Eli Lilly, Janssen Pharmaceuticals. DT Rubin: grant support from Takeda; and has served as a consultant for AbbVie, AltruBio, Apex, Avalo Therapeutics, Bristol Myers Squibb, Buhlmann Diagnostics Corp, Celgene, Connect BioPharma, Intouch Group, Iterative Health, Janssen Pharmaceuticals, Lilly, Pfizer, Samsung Neurologica, and Takeda. He serves on the Board of Trustees for the Crohn's & Colitis Foundation and is on the Board of Directors for Cornerstones Health.

Informed consent was obtained for this case report.
